# Adolescents’ Experiences of Cyber-Dating Abuse and the Pattern of Abuse Through Technology, A Scoping Review

**DOI:** 10.1177/15248380241227457

**Published:** 2024-02-07

**Authors:** Rojan Afrouz, Sevi Vassos

**Affiliations:** 1 Deakin University, Geelong, VIC, Australia

**Keywords:** cyber-dating abuse, intimate partner violence, technology-facilitated sexual violence, adolescents, digital technology, social media

## Abstract

While the proliferation of online social platforms has become a significant part of virtual interactions between intimate partners, digital technology has also created the conditions for increased control and abuse, which is known as “cyber-dating abuse,” a technology-facilitated form of intimate partner violence. This paper reports a scoping review of qualitative studies to explore the patterns, nature, and consequences of cyber-dating abuse among young people and how digital technology influences dating abuse. Several databases were searched to find relevant papers, including EBSCOhost, Scopus, SocINDEX, ProQuest, Taylor and Francis Online, PubMed, and Google Scholar. All peer-reviewed papers that used qualitative and mixed methods exploring cyber-dating abuse since 2010 were scanned, and 23 papers were included in this scoping review. Thematic analysis was employed to analyze the data. Findings showed that online platforms and digital technology have potentially exacerbated the monitoring, control, and surveillance of young women, often by young men. This scoping review also found a mixed report of gender-based victimization in relation to cyber-dating abuse; however, girls were more likely to face severe and negative consequences compared to boys. Gender-based societal norms and associated behavioral and social factors may increase the risk of cyber-dating abuse among young women. The scoping review reinforces the importance and value of preventative and early identification strategies in young people’s school-based education, with a sharp focus on violence and abuse in the online space, respectful relationships, and informed consent in intimate relationships.

## Introduction

Intimate partner violence (IPV), including technology-facilitated abuse, is prevalent worldwide (Afrouz, 2021; Sardinha et al., 2022; Wake & Kandula, 2022). IPV involves actions and behaviors that inflict physical, psychological, or sexual harm against a person in the current or previous relationship (Rajah & Osborn, 2020, p. 1373). In its most recent study of IPV, the World Health Organization (WHO, 2014) found that approximately 50% of girls and young women aged 15 to 24 had experienced IPV. The experiences of IPV have significant impacts on women’s physical and mental health throughout their lifetime (Afrouz, 2021; Lundgren & Amin, 2015). Furthermore, IPV studies show that adolescents and young adults are at higher risk than other age groups of experiencing sexually based IPV where digital technologies are used to perpetrate harmful sexual acts (Henry & Powell, 2018; Lundgren & Amin, 2015; Stöckl et al., 2014)

“Cyber-dating abuse” is the term often used in IPV research to describe the role of technology-facilitated abuse among young people. Cyber-dating abuse consists of “the control, harassment, stalking, and abuse” against an intimate partner via digital technology and online platforms (Zweig et al., 2014, p. 1306). Some forms of cyber-dating abuse include psychological aggression, control and monitoring behavior, cyber harassment, manipulation, image-based sexual violence, and spreading rumors or humiliating multimedia content (Rodríguez-deArriba et al., 2021). While cyber-dating abuse is the most used term, other terms have also been used across literature, including digital dating abuse, cyber aggression in relationships/dating, cyber-dating violence, and partner cyber abuse (see Caridade et al., 2019, p. 162). There is emerging evidence that victims of cyber-dating violence are more likely to experience other forms of IPV (Dick et al., 2014; Temple et al., 2016; Zweig et al., 2013). For example, a longitudinal study by Lu et al. (2021) found that cyber-dating abuse is predictive of in-person IPV. Lu et al. (2021) concluded that young people’s awareness of the sign of cyber-dating abuse could potentially mitigate the risks of associated in-person abuse. Nevertheless, the relationship between cyber-dating violence and in-person violence remains nebulous and complex (Sen, 2016).

The existing research indicates that digital tools and online platforms have potentially increased violence against women and sexual violence, particularly in intimate relationships (Afrouz, 2021; Flynn et al., 2021; Hertlein, 2021; Powell & Henry, 2019). Digital technology has become a preeminent medium of communication and facilitated romantic relationships for adolescents and young people, resulting in that the nature and context of personal relationships might have changed (Lu et al., 2021). Notably, generations Y and Z (Millennials and Centennials) exhibit a greater tendency to use smartphones, which allow young people constant access to the internet and social media (Linares et al., 2021; Temple et al., 2016), increasing the risk of dating violence through digital technology among young people. Nevertheless, the scope and nature of cyber-dating abuse are yet to be fully understood. To this end, this paper reports on the findings of a scoping review of qualitative studies on cyber-dating abuse literature. The aim was to map and synthesize existing research systematically, with a view to scoping the issue and establishing the research gap through which future research directions and intervention strategies can be adapted in the increasingly digital society.

## The Prevalence and Impacts of Cyber-Dating Abuse

Reports on the prevalence of cyber-dating abuse are still emerging, and the existing data are inconsistent due to methodological or measuring differences (Caridade et al., 2019; Lu et al., 2021). Nevertheless, there is a growing body of research on cyber-dating abuse, albeit concentrated in North American or European countries. For example, Zweig et al.’s (2014) U.S. study reported that 28% of middle and high school students experienced cyber-dating abuse. Additionally, Borrajo et al.’s (2015) study of Spanish college students shows that 50% had an experience of cyber-dating abuse within the last 6 months. Furthermore, a large-scale Italian study (*n* = 1,405) found that 64% have been victims of at least one form of cyber-dating abuse (Morelli et al., 2018). Despite these reports, most of the existing research has predominantly focused on the perpetration of cyber-dating abuse rather than abuse patterns, the victims’ lived experiences, and risk factors (Caridade & Braga, 2020).

Adolescence, the life stage between 10 and 19 years (UNICEF, 2022), is the most significant developmental period in a person’s life cycle (Reyes et al., 2016). Borrajo et al.’s (2015) study found that younger adolescents were more likely to experience cyber-dating abuse. Young people, particularly young women, may not recognize signs of IPV in cyber-dating, resulting in misinterpreting abusive online behaviors as a sign of love or attention (Lu et al., 2021; Towler et al., 2020). At the same time, young people’s, particularly young women’s, responses are critical because these can often evolve into a life-long pattern of response to abuse.

Cyber-dating abuse is one of the most common forms of abuse against adolescents (Murray, 2019). A potential for anonymity, invisibility, and capacities of digital platforms for asynchronous communications might create the conditions for increased dating violence through digital technology (Lu et al., 2021). In addition, the spaceless nature of online harassment and threats potentially reduces victims’ capacity to escape such violence and abuse (Afrouz, 2021; Harris, 2018; Lu et al., 2021). Cyber-dating abuse is identified as a distinct form of abuse because it transcends geographical boundaries. Dating abuse has significant consequences on young people’s mental health, physical health, and social relationship (Murray, 2019). Furthermore, Murray (2019) warned that dating abuse victimization could be a significant risk factor for teen suicide, particularly among young women. Yet, the harm and the well-being impacts of this type of abuse are just as potent as any other form of IPV.

It is notable that many large-scale quantitative studies did not focus on the severity, duration, or impacts of cyber-dating abuse. As such, these studies can only provide a partial picture in which potential gender-specific and in-depth understanding of young people’s experiences are largely hidden (Brown et al., 2020). A review of the research base reveals inconsistent findings regarding the gendered nature of cyber-dating abuse. For example, a Canadian study reported that out of 190 teenagers, 35.8% experienced cyber-dating abuse, and there were no notable gender differences in the prevalence rate (Smith et al., 2018). Taylor and Mumford (2016) found that while there were no gender differences in victimization, girls were more likely to be the perpetrators of cyber-dating violence. Studies focusing on the lived experience of cyber-dating abuse could provide a more nuanced picture of gender differences. For example, Reed et al.’ s (2017) study reported that while both young girls and young boys experience cyber-dating abuse, the consequences and impacts of cyber-dating violence are more harmful to girls. Young women were more likely to experience the negative impacts of cyber-dating abuse; therefore, it can be argued that the gender lens should be applied in designing research and data analysis (Brown et al., 2020).

Furthermore, little is known about how adolescents understand abusive behavior; some behavior might be normalized through digital technology (Towler et al., 2020). In the digital age, young people often connect online with people they also know in person. Sen (2016) found that young people were more likely to experience abusive behavior from people they already knew. Therefore, it might be confusing for adolescents to distinguish when romantic engagement becomes abusive monitoring and controlling in the online environment (Temple et al., 2016). It can be concluded that cyber-dating abuse is a prominent social issue with severe well-being impacts among adolescents, even though its prevalence and nature are yet to be fully understood.

In light of the above, this review sought to privilege research on the patterns, nature, and consequences of cyber-dating abuse among young people. The aim was to synthesize the findings of qualitative studies conducted in the last 13 years with a view to gaining insights into young people’s experiences of youths’ cyber-dating abuse, its patterns, nature, and consequences and how digital technology has become a tool for dating abuse.

## Methodology

A scoping review was conducted to summarize the existing literature and define gaps in the current research base within a short period of time (Arksey & O’Malley, 2005; Pham et al., 2014). Arksey and O’Malley’s (2005) scoping review process was used to guide the review. Arksey and O’Malley (2005) articulate five stages in the scoping review process: (a) developing research questions; (b) identifying studies based on inclusion criteria; (c) selecting literature; (d) data extraction and recording the data; and (e) and summarizing results.

### Developing Research Questions

The scoping review questions were developed to explore the youths’ experiences of cyber-dating abuse, including the nature, impacts, and types of cyber-dating abuse. In line with the abovementioned research aims, the review was conducted to respond to the following key questions: How did young people experience cyber-dating abuse based on the existing qualitative research? What are the patterns, nature, and consequences of cyber-dating abuse through digital technologies and online platforms? What are the gender-based differences in young people experiences of cyber-dating abuse?

### Identifying Relevant Studies

Subsequently, based on the subject coverage, relevance to the research topic and researchers’ access the following databases and platforms were searched to find relevant studies: EBSCOhost, Scopus, SocINDEX, ProQuest, Taylor and Francis Online, PubMed, and Google Scholar. The review included peer-reviewed journal articles and book chapters exploring young people’s (aged 10–24) experiences and perceptions of cyber-dating abuse. Based on the research questions, the following key terms and keywords were identified: cyber-dating abuse, cyber-dating violence, and online dating abuse within an intimate relationship. All search terms used are outlined in Table 1.

**Table 1.** Keywords Used to Identify Articles.

Search (S)	Keywords
S1	Technology-facilitated dating abuse
S2	Digital dating abuse
S3	Cyber-dating abuse
S4	Technology-facilitated coercive control
S5	Revenge porn
S6	Online abuse
S7	Youth dating abuse
S8	Teen dating violence
S9	Image-based abuse
S10	S1 OR S2 OR S3 OR S4 OR S5 OR S6 OR S7 OR S8 OR S9
S11	Intimate partner violence
S13	Domestic violence
S14	Battered women
S15	Domestic abuse
S16	S11 OR S12 OR S13 OR S14 OR S15
S17	Intimate Partner
S18	Romantic relationship
S19	S10 AND S16 AND S18
S 20	Adolescents
S 21	young adults
S 22	Teens
S 23	Youth
S 24	S10 AND S16 AND S18 And S 19

In addition to identified qualitative studies, the review considered “qualitative-dominant mixed method” studies (Johnson et al., 2007) as well as existing qualitative literature reviews. The authors decided to include papers published later than 2010. This timeframe was selected because of significant changes in digital technology and online social media on people’s relationships and communications since 2010 (Afrouz, 2021).

Inclusion and exclusion criteria: the review focused on cyber-dating abuse among adolescents within a romantic or intimate relationship. All qualitative and mixed papers that focused on IPV and partner abuse through digital technologies or online platforms were included if they focused on young people’s (aged 10–24) experiences. Studies reporting on the experiences of practitioners and/or parents were excluded. Studies that reported dating abuse without looking at the role of digital technology and digital platforms were excluded. Papers that only focused on online harassment and sexual assaults and cyberbullying and online misogyny outside of an intimate relationship were also excluded. Additionally, papers that only focused on the benefits of digital technology in seeking help for cyber-dating abuse were excluded as this was beyond the scope of the research questions. Quantitative studies were excluded as these mainly focus on measuring incidence and correlations rather than exploring experiences of cyber-dating abuse and patterns of behaviors.

### Literature Selection

**Figure 1.**
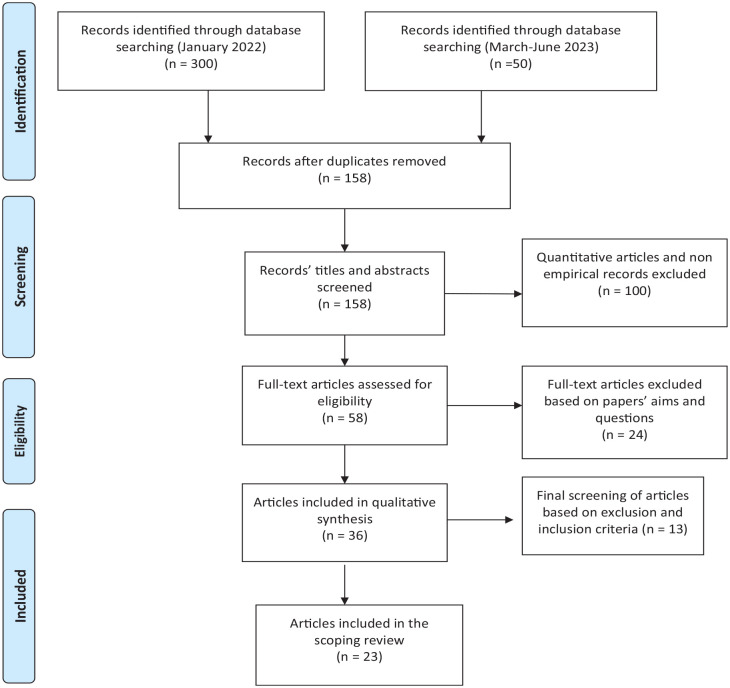
Process of literature selection.

In the first step of this review, the first author scanned titles and abstracts of the retrieved papers. The first round of paper selection was completed based on the study abstracts. Thirty-four papers were included in the full-text scanning, and 23 papers were included based on the inclusion/exclusion criteria for this scoping review, as both authors agreed on (Figure 1) and (Table 2). Although a systematic quality assessment is not required for scoping reviews ( Grant and Booth (2009), the CASP (Critical Appraisal Skills Programme, 2017) was adopted to evaluate the overall quality of the methodological clarity, study design, ethical consideration, and process of data collection as part of the paper the selection process. No study was excluded based on CASP evaluation. Only peer-reviewed studies were included in the review in line with the quality assessment.

**Table 2.** Summary of Articles Included in the Review (*n* = 23).

Author/s (Year)	Country	Design	Sample/s (*n*)	Age Group	Instrument/s
Alsawalqa (2021)	Jordan	Qualitative	104 females	Undergraduate with majority18–20 years	Interview
Baker and Carreño (2016)	USA	Qualitative	8 Focus groups 39 (female and male)	14–19 years	Focus groups
Baker and Helm (2010)	USA	Qualitative	Focus groups 51 (female and male)	13–19 years	Focus groups
Belotti et al. (2022)	Italy	Qualitative	7 Focus group 43 Adolescents (75.6% vs. 24.4% of male students)	14–19 years	Focus groups
Brown et al. (2020)	Australia	Qualitative	4 Focus groups *N* = 38 (female and male)	16–24 years	Focus groups
Draucker and Martsolf (2010)	USA	Qualitative	56 (female and male)	18–21 years	Interview
Fernet et al. (2023)	Canada	Mixed methods	14 interviews	14–25 years	Interview
Goldstein and Flicker (2020)	Canada	Qualitative	25 (24 female and/or queer/non-binary, and one male	Teenagers (The average age = 16.7)	Focus groups
Hellevik (2019)	Norway	Qualitative	21 (female and male)	15–18 years	Interview
Howard et al. (2019)	USA	Qualitative	70 Females	15–18 years	Interview
Kulkarni et al. (2019)	USA	Qualitative	9 Focus groups *N* = 86 (All genders)		Focus groups
Lachapelle et al. (2021)	Canada	Mixed methods	16 (female and male)	15–17 years	Interview
Lucero et al. (2014)	USA	Qualitative	4 Focus groups *N* = 23 (female and male)	High school and sophomores’ age	Focus groups
Mandau (2021)	Denmark	Qualitative	157 posts written by females on the counseling hotline	11–20 years	Online posts
Reed et al. (2020)	USA	Mixed methods	262 (Female, male and other gender identiﬁcation)	14–18 years	Survey/open-ended questions
Rueda et al. (2020)	USA	Qualitative	Interview with staff *N* = 12 Focus groups with teenagers *N* = 13	14–22 years (participants of FG)	Interviews and Focus groups
Rueda et al. (2015)	USA	Qualitative mixed methods	20 Focus group (*N* = 64) and a videotaped observations of couples (*N* = 34 couples) (female and male)	15–18 years	Focus groups and observations
Stanley et al. (2018)	Bulgaria, Cyprus, England, Italy, Norway	Mixed methods	4,564 completed survey and 91 interviews	14–17 years	Survey/interview
Stonard (2020)	UK	Qualitative	9 Focus groups *N* = 54 (female and male)	13–16 years	Focus groups
Stonard et al. (2017)	UK	Qualitative	8 Focus groups 52 (female and male)	12–18 years	Focus groups
Van Ouytsel et al. (2019)	Belgium	Qualitative	4 Focus groups *N* = 55 (female and male)	15–18 years	Focus groups
Weathers et al. (2019)	USA	Qualitative	10 Females	18–24 years	Interview
Weathers and Hopson (2015)	USA	Qualitative	10 Females	18–24 years	Interview

### Data Extraction and Data Analysis

Both authors read the full text of included papers for familiarity with the data. The scoping review was informed by feminist standpoint epistemology that values women’s unique insights into the world (Hesse-Biber & Leavy, 2011). Our primary objective was to uncover and center young people’s experiences along with a gender lens to elucidate understanding of cyber-dating abuse from a feminist perspective. In line with Brooks and Hesse-Biber (2011), the voices and experiences of girls and women were privileged to counteract the historical trend of excluding and/or misrepresenting women’s lives and perspectives in the construction of knowledge.

The initial plan was also to compare studies that focused on young people under and over 18; however, the comparison was not possible given the use of different age brackets across individual studies. Therefore, both authors agreed to include all papers that focused on adolescents and young adults (aged 10–24) and analyzed the data thematically from a feminist standpoint without comparing findings for specific age brackets. All included papers were uploaded to a shared NVIVO 14 (Version 14.23.1) file, a qualitative research software program (see https://www.qsrinternational.com/) to manage, synthesize, and analyze data. Drawing on Braun and Clarke (2019) as a guide, the first author started the inductive thematic analysis by reading line by line the results section of all included papers to familiarize herself with the data, highlight relevant phrases and words, develop initial coding, and make first-hand themes. Then, both authors reviewed identified themes and regrouped and refined these with an emphasis on uncovering gender differences. In addition, a feminist standpoint epistemology enabled us to sharpen our focus on identifying the power structures and cultural norms that perpetuate young women’s and girls’ subordination. Differences and similarities have emerged in the reported experiences of young women and young men across each theme. Five overarching themes with 12 subthemes emerged from the analysis, outlined in the finding section. A summary of critical findings is presented in Table 3.

**Table 3.** Summary of Critical Findings.

Technology and online platforms have become new tools for control and abuse, which made cyber-dating abuse more elusive and perplexing.
The most common forms of cyber-dating abuse were monitoring and control, image-based abuse, harassment, and coercion.
Anonymity and the spaceless nature of online abuse, particularly after the relationship finishes, were precipitating (risk) factors contributing to control and abuse through digital tools.
Some young people have approached password sharing to show they are trustworthy, resulting in control and online abuse.
Some young people, particularly young women, experienced isolation, self-blame, and self-censorship following their experiences of cyber-dating abuse.
Young women were more likely to experience consequences of cyber-dating abuse, such as public humiliation and harm to reputation and blame.
Help-seeking for cyber-dating abuse has remained challenging, and many young people, particularly young women, preferred accessing informal support when experiencing cyber-dating abuse.

## Results

Twenty-three papers met the inclusion criteria for this review. Interestingly, only one of the included papers reported on cyber-dating abuse in a non-Western country, namely Jordan. Unsurprisingly, all other papers reported on the phenomenon of cyber-dating abuse in the Western world, with the majority of papers from North America, more specifically, 11 papers from the United States and three papers from Canada. The review uncovered a further seven papers reporting on cyber-dating abuse in European countries, one of which was a transnational European study, and one paper from Australia.

Study sample sizes also varied considerably. One-third of the included studies (8 out of the 23) had a relatively small sample of 10 to 30 participants. Just under half of the included studies (11 out of 23) had a sample size of 30 to 100 participants. A further 4 papers had a sample base of more than 100 participants, with the transnational European study as the largest of all the studies with 4,564 participants.

In terms of data collection methods, just over half of the included studies (13 out of 23) involved focus group interviews, seven studies used individual interviews, one study relied on online posts, and only one study, the largest of the studies, involved surveys.

### The Context of Cyber-Dating Abuse

#### The Elusive Nature of Dating Abuse

The included papers reported that cyber-dating abuse is prevalent and insidious. Cyber-dating abuse has been described as deliberate abuse, harm, and a hurtful game without informing the victim (Goldstein & Flicker, 2020). Youth who participated in the study by Stonard (2020) described cyber-dating abuse as an invasion of privacy, making controlling, humiliating, monitoring, and insulting a partner easier through digital technology. The perpetrators could constantly abuse their victims from a distance and beyond a physical location. In addition, the participants in the study by Alsawalqa (2021) defined this form of abuse as aggression, threats, and abuse.

Young people often experienced cyber-dating abuse as elusive, ambiguous, and incongruous with other forms of abuse (Howard et al., 2019; Lucero et al., 2014; Weathers et al., 2019). This, in turn, often resulted in feeling overwhelmed, heightened levels of (dis)stress, and an accompanying sense of powerlessness. Notably, male adolescents (age 12–15) in the Weathers et al.’s (2019, p. 339) study were more likely than their female counterparts to report that they could stop any presenting online dating abuse by ignoring it.

Interestingly, there were mixed understandings of what was and was not acceptable behavior in cyber-dating (Belotti et al., 2022). For instance, Baker and Helm (2010, p. 163) found that young people frequently conceptualized abuse over social media “as a dating problem, but not as dating violence.” Particularly, it was difficult for young people to discern the point at which online dating behaviors became annoying and/or obsessive (Stonard et al., 2017). Furthermore, Howard et al. (2019) found that some young people did not perceive online dating abuse to be as harmful as other forms of abuse, primarily because they felt they had the choice to disengage from the online space. Yet, these participants identified that social media could be harmful due to the permanent digital footprint maintained in the online environment (Howard et al., 2019, p. 8).

#### Tech Has Become a Tool for Abuse

Digital technology and online platforms have become tools to facilitate abusive acts (Draucker & Martsolf, 2010; Rueda et al., 2020; Stonard et al., 2017). Young women and girls in the study by Stonard et al. (2017) reported that digital technology and online platforms provided more opportunities for abuse because it is often hidden, while at the same time harder to address or prevent; as a 13-year-old female noted, “yeah, they can, they’ll always find a way to get to you” (Stonard et al., 2017, p. 2102).

Rueda et al. (2015) and Baker and Carreño (2016) also highlighted that the availability of information and communication technologies enabled perpetrators to easily insert themselves into their victims’ life without needing to make direct contact. Behaviors such as “insisting on communication at all times” enabled control over and isolation of partners (Rueda et al., 2015, p. 434). Notably, one male participant in the study conducted by Stonard (2020, p. 5) claimed that “technology was designed for this” because of its availability and easy access (Stonard, 2020). Mandau’s (2021) participants also highlighted that the affordability and accessibility of online communication platforms and the dynamics of intimate relationships probably created the conditions for digital technology to be used as a tool for abuse (Mandau, 2021). Furthermore, the public nature of online spaces can escalate abusive behaviors and significantly harm victims’ social and emotional well-being (Rueda et al., 2020). For example, in more traditional and/or religious communities, sharing an intimate photo online was particularly damaging for young women in Jordan (Alsawalqa, 2021; Stanley et al., 2018).

### Types of Cyber-Dating Abuse

Studies identified different forms of cyber-dating abuse, including monitoring (Brown et al., 2020; Lucero et al., 2014; Reed et al., 2020; Stonard et al., 2017; Van Ouytsel et al., 2019), image-based abuse (Belotti et al., 2022; Stonard, 2020), and sexual coercion and sexual humiliation (Hellevik, 2019; Reed et al., 2020). Alsawalqa (2021, p. 5) also identified the following as various forms of cyber-dating abuse:

posting, uploading, or sharing sexual or personal photos online, or recording romantic phone calls, video chats, or conversations on social media, as well as sexual and amorous talk to embarrass the victim and blackmail them for money, sexual exploitation, or manipulation.

#### Monitoring and Controlling Partners Over Social Media

Several studies identified online monitoring behaviors, such as checking a partner’s online and/or phone activities, as the most prominent form of cyber-dating abuse (Brown et al., 2020; Lucero et al., 2014; Reed et al., 2020; Stonard et al., 2017; Van Ouytsel et al., 2019). The accessibility of online platforms was again highlighted as an enabler for creating fake online profiles (Hellevik, 2019; Rueda et al., 2015) and controlling the victims’ social media accounts (Baker & Helm, 2010; Hellevik, 2019), as digital instruments and social media forums normalized controlling and monitoring (Belotti et al., 2022).

The included papers also highlighted some gender differences regarding monitoring behaviors. Brown et al. (2020) and Lucero et al. (2014) found that young women were more likely to intermittently monitor their partners’ text messages or online interactions as the opportunity arose. On the other hand, young men were more likely to use devices to systematically track their partner’s activities, thus creating a sense of omnipresence. For instance, one18-year-old female in Hellevik’s (2019) study described, “I had to stop hanging out with male friends. . .[or] be friends with boys on Facebook, those he [boyfriend] knew who [they] were, but I was not allowed to talk to them” (Hellevik, 2019, p. 182). Notably, boys who participated in the Baker and Carreño (2016) study reported that monitoring behaviors were necessary to maintain and control their relationships.

#### Image-Based Abuse and Unwanted and Coercive Sexting

Images could be easily shared through online platforms and social media for abusive purposes (Belotti et al., 2022; Stonard, 2020), resulting in feelings of helplessness, particularly for young women (Weathers & Hopson, 2015). Whether images were created with consent or not, an intimate photograph could be used after the relationship ended to threaten, intimidate, humiliate, and/or harass the former partner (Hellevik, 2019).

Young men were more likely to share their partner’s photographs with their peers, potentially causing fear, anxiety, and breaches in safety and privacy for young women. On the other hand, young women were less likely to share their partners’ photographs with third parties, as young women were often sensitive to the issues of sharing images without consent (Brown et al., 2020). As articulated by one young woman in Brown et al.’s (2020) study,

I’m sure most of us wouldn’t go and say, “Here’s a picture that my boyfriend sent me, or this guy sent me. . .Isn’t he great?” Maybe we feel like it happens to us, and we know how that feels, and we’re not taught to be sexually domineering or sexually open (p. 6).

Young women were more likely to be subjected to young men sharing their sexual photos and/or young men taking sexual images without informed consent (Mandau, 2021; Weathers & Hopson, 2015). While both genders shared their photos, the act of taking screenshots without permission was exclusively a behavior perpetrated by boys (Mandau, 2021).

In the study conducted by Lucero et al. (2014), young men reported that it was common to show “sexting” messages exchanged with their girlfriends to their male friends. However, for young women and girls, sexting was more likely to be maintained as a private conversation. Pressure to engage in sexting was often experienced as a form of cyber-dating abuse (Reed et al., 2020), and unwanted texts caused harm, particularly to young women (Hellevik, 2019).

#### Coercion, Harassment, and Other Forms of Online Aggression

Hellevik’s (2019) and Reed et al.’s (2020) studies identified sexual coercion and sexual humiliation as forms of cyber-dating abuse. Some young women were coerced to share their photographs, regardless of their sexual nature (Reed et al., 2020). Furthermore, Howard et al.’s (2019) study identified online stalking as a distinct form of cyber-dating abuse (Howard et al., 2019). Emotional abuse, verbal aggression, and threatening messages were also common forms of cyber-dating abuse (Alsawalqa, 2021; Baker & Carreño, 2016; Reed et al., 2020; Stonard et al., 2017). Draucker and Martsolf (2010) indicated the diversity of cyber-dating abuse and its inextricable links to other forms of physical and sexual violence.

### Precipitating (Risk) Factors

#### Risk Factors in Cyber-Dating Abuse

Anonymity through online technology makes breaching privacy easier, and the abusive partner can readily obtain information from others through fake accounts (Stonard, 2020). Anonymity is now more viable through digital technology, particularly as the potential to meet in person was limited at the time of COVID-19 (Goldstein & Flicker, 2020). Furthermore, the nature of online communications increases the likelihood of misinterpretation, misunderstandings, and arguments (Rueda et al., 2015). Communications and interactions between intimate partners can be publicly available over social media platforms, blurring the line between private and public domains. This can become a risk factor that could result in social and personal implications for victims, making it difficult for victims to discern the abuse immediately (Stonard, 2020).

The spaceless nature of online abuse that continues after an intimate relationship end might result in stress and confinement, and perpetrators continue to exert power (Weathers et al., 2019). The ongoing pressure and the unlikelihood of removing the perpetrators within the online world might suppress women’s emotions and thoughts (Baker & Carreño, 2016; Weathers et al., 2019). Stonard et al.’s (2017) study found that young women experienced online abuse as potentially worse than in-person abuse, particularly more challenging when the relationship ends. The fact that abusive activities and messages remain in online spaces “stays with you” and “gets into your head” (Stonard et al., 2017, p. 2104)

#### Password Sharing and Privacy Issues

Password sharing was not uncommon among participants, especially if the partner requested it (Lucero et al., 2014; Van Ouytsel et al., 2019). Young women often shared their passwords with their partners as an expression of trust in the relationship (Belotti et al., 2022; Fernet et al., 2023; Kulkarni et al., 2019) or to prove that they had nothing to hide (Lucero et al., 2014; Rueda et al., 2015). While password sharing was identified as a way of spying, controlling, and monitoring by some boys and girls, young men had a more cautious approach to password sharing (Lucero et al., 2014). Boys perceived password sharing as a violation of their privacy. Interestingly, Rueda et al. (2015) found that while password sharing was often considered acceptable behavior, checking each other’s phones was considered monitoring (Rueda et al., 2015).

#### The Role of Jealousy

Young people identified jealousy and suspicions as a cause of cyber-dating violence (Rueda et al., 2015; Van Ouytsel et al., 2019), resulting in mistrust and surveillance (Rueda et al., 2015). Lucero et al. (2014) and Baker and Carreño (2016) found that jealousy was the main reason for monitoring. As the relationship and online activities can be monitored all the time by a partner or inappropriate use of digital technology, any move, such as liking a photograph, can be a cause of jealousy and, therefore, might trigger online abuse (Baker & Carreño, 2016). Nevertheless, monitoring an intimate partner online was perceived as endearing by some participants in the study conducted by Fernet et al. (2023), which can be seen in the following quote: “I think it is cute anyway. I do not find it (their partners access of their online interactions) to be too much” (p. 305).

### Victimization and Responding to Cyber-Dating Abuse

#### Gendered Experiences of Victimization and Perpetration of Cyber-Dating Abuse

Brown et al. (2020) and Alsawalqa (2021) found that although both genders used digital technology to control and monitor their partners, young women were more likely to be the victims. Traditional gender norms and attitudes resulted in more negative consequences for young women. As abusive messages and materials also remain online, the impacts on female victims are ongoing (Stonard et al., 2017), often resulting in self-isolation (Baker & Carreño, 2016). Online sexual harassment was also more likely to result in feelings of fear among young women (Stonard, 2020). The harm to young women victims was further exacerbated as their responses to the abuse were more likely to be characterized as irrational, naive, and emotional (Alsawalqa, 2021).

Reed et al.’s (2020) study found that although both genders were unsure of certain abusive behaviors, young men were more likely to commit digital sexual abuse. In the study by Mandau (2021), young girls blamed themselves for sharing their photos with their partners, describing themselves as stupid and naive. Goldstein and Flicker (2020, p. 70) found that following the COVID-19 pandemic restriction, teen girls were concerned that the online environment might further reinforce “gendered relations of male agency-female submissiveness” as a male partner could connect when they wanted and disappear to a period of time if needed.

#### Help-Seeking Behaviors and Coping Mechanisms

Young women were less likely to disclose their experiences to others, including their parents (Howard et al., 2019). Some girls tried to avoid or ignore the abuse as a mechanism to stop it (Stonard et al., 2017). However, blocking the abuser could lead to frustration and further harassment by the abuser (Baker & Carreño, 2016).

The participants in the study by Alsawalqa (2021) found that police were helpful for victims/survivors in removing online abuse. However, young women in the study by Alsawalqa (2021) were less likely to seeking support due to shame and the fear of family punishment or being blamed and ridiculed. Thus, informal support and trusted people were significant in young women’s experiences of dealing with online dating abuse (Alsawalqa, 2021; Lachapelle et al., 2021; Weathers et al., 2019). For instance, young women in Jordan preferred seeking emotional support from their informal networks (Alsawalqa, 2021), and if they asked for police, they often got support privately due to the fear of victim blaming. Nevertheless, participants in the study conducted by Weathers et al. (2019) felt that their family and friends did not often recognize the severity of online abuse, as one participant articulated, “I didn’t know what to do, I was younger, and no one else understood how creepy it was. Um, so . . . I don’t know. Just. freaked me out a lot” (Weathers et al., 2019, p. 337).

### Impacts of Cyber-Dating Abuse

#### Harmful Impacts on Victims’ Health and Well-Being

Cyber-dating abuse impacts young women in several ways, including emotional effects, anxiety, stress, depression, and social and physical impacts (Alsawalqa, 2021). There were also social impacts, such as public humiliation and harm to reputation (Reed et al., 2020). While cyber-dating abuse was harmful to all young people, Brown et al. (2020) found that the consequences of abuse were more severe against young women in Australia. Alsawalqa’s (2021) findings in Jordan also indicated that young women were more vulnerable and experienced consequences of eating disorders or attempted suicides.

Some abusive online acts result in offline forms of abuse (Draucker & Martsolf, 2010; Hellevik, 2019; Lachapelle et al., 2021; Stonard, 2020), or victims could possibly experience both (Rueda et al., 2020; Stanley et al., 2018). Participants in the study conducted by Hellevik (2019) reported that there was an interplay between online and offline abuse; more specifically, online abuse could be a threat to physical abuse.

#### Isolation, Self-Blame, and Self-Censorship

Brown et al.’s (2020) study found that young women were more likely to experience the harmful impacts of digital technology, including feelings of being trapped, insecure, and isolated. Alsawalqa’s (2021) study identified that young women were often blamed for engaging in unacceptable online activities and, therefore, responsible for the advent of online dating abuse. Additionally, Mandau (2021, p. 439) found that young women experienced “turning inwards” with a range of negative emotions and self-blame for seemingly avoidable experiences of imaged-based online abuse. Self-blame was often accompanied by a reluctance to seeking help.

Many participants in the study conducted by Draucker and Martsolf (2010) did not want to “deal with” harassment and threats online and therefore decided to either limit or disengage from online activities. Young women often self-censored, socially isolated, and refrained from using social media in response to cyber-dating abuse (Baker & Carreño, 2016; Weathers et al., 2019). Van Ouytsel et al.’s (2019) study found that young women were less likely to post bikini-clad photographs of themselves online if their boyfriends disapproved. Despite this, self-censorship often resulted in further online abuse (Weathers & Hopson, 2015).

## Discussion

The overall findings of this scoping review highlighted that digital technology and tools have provided new avenues and possibilities for cyber-dating abuse. However, the experiences of young people in the reviewed studies indicated that cyber-dating abuse was still confusing, ambiguous, and perplexing, resulting in being taken less seriously by young people or their informal support. Thus, our findings indicated that while the consequences of cyber-dating abuse were harmful to young people, particularly young women, they were less likely to identify and resist the early signs of abuse (e.g., password sharing, jealousy, and monitoring). Cyber-dating abuse can be confusing, and some young women and girls may not recognize when the relationship becomes violent. Nevertheless, there was a gap in the literature on how relevant education for young people, particularly girls and young women, could help them identify the signs of cyber-dating violence and how they should be reported when needed. While traditional forms of dating abuse can be overt and observable, the unique features of digital technology, and the complex nature of online platforms and social media, including anonymity and moving beyond physical locations, contributed to the sense of uncertainty around which behaviors were appropriate and created challenges in identifying and addressing cyber-dating abuse in the virtual space.

The feminist standpoint epistemology in the analysis of included papers enabled authors to gain useful insights into the gendered nature of cyber-dating abuse. This review’s findings contrast with some quantitative and large-scale studies (see Peskin et al., 2017; Smith et al., 2018; Taylor & Mumford, 2016; Temple et al., 2016) that reported no significant gender difference in cyber-dating prevalence. This review suggests that online abuse often mirrors the male power and the gendered nature of abuse in an offline world among young people, as abuse is more likely to be perpetrated by a male against their female partner (Afrouz, 2021; Williams et al., 2023). Notably, this review found that although both young men and young women engage in cyber-dating abuse, the research suggests gender-based differences in monitoring and control. Also, men were more likely to exert their power by being omnipresent, violating privacy, and monitoring and controlling victims in a continued and systematic way. This review confirms a pattern of hierarchical and systematic gender dominance in which young men use power and abuse to claim power in cyberspace.

In addition, young boys were the dominant power in social media platforms to isolate their partner socially, and they were more likely to humiliate their intimate partner. On the other hand, female victims were more likely to reduce their public activities, censoring and blaming themselves, and hence, they were less likely to seek help. This finding mirrors the research findings on adult women’s experiences of Technology-Facilitated Domestic Abuse (TFDA). Humiliation following disempowerment by an abusive partner resulting in the victim’s disconnection from the online world is widely reported in the TFDA research literature (Afrouz, 2021; George & Harris, 2014).

Furthermore, feminist theories provide an explanation for how traditional gender roles and associated gender-based structural inequalities can precipitate and perpetuate IPV (Cocker & Hafford-Letchfield, 2022). From a feminist perspective, male partners are more likely to assume a power position within their intimate relationships, and violent behavior becomes a means for maintaining domination and power over their partner (Reyes et al., 2016). As there is general agreement among feminist writers that gender inequality is a driver of IPV among young people, as in the adult population (Lundgren & Amin, 2015), this study’s findings can support some heteronormative gender roles and attitudes, namely women as subordinate and men as dominant in decision-making, are significant risk factors in cyber-dating abuse among adolescents, as these are reinforced in the online space (Reyes et al., 2016). Attitudes and behaviors that reflect gender inequality and sexism can reinforce cyber-dating abuse (Fernández-Antelo et al., 2020). In particular, Reyes et al.’s (2016) study identified that traditional gender roles and attitudes, as well as the widespread acceptance of certain [abusive] behaviors in the online space, were key factors contributing to the dating abuse perpetration; although the data indicated a degree of uncertainty in conclusion, which needs to be explored in the future research. In addition, as Powell et al. (2018) indicated, the existing research is overwhelmingly focused on cisgender and heteronormative perspectives on cyber dating abuse. Consequently, the findings of this review reflect a narrow focus on heteronormative gender identities and sexual orientations, which do not adequately account for the diversity of experiences of LGBTI+ communities.

Furthermore, young victims face a degree of uncertainty in abusive relationships, blaming themselves and taking responsibility for their experiences of cyber-dating abuse. Kennedy and Prock (2018) found that self-blame is often one of the consequences of sexual assault among survivors, which could be a significant barrier to seeking help. They also indicated that people across diverse groups (e.g., women, migrants, LGBTIQ+, and religious minorities) were more likely to experience shame and self-blame. Self-blaming can also be linked to the way in which society tends to pass judgment on women, often holding them responsible for sexual abuse (Flynn et al., 2022). Flynn et al. (2022) further argue that victims’ self-blame can be a result of internalizing societal attitudes and the existence of sexual double standards, which might also act as a potential barrier to seeking help. As indicated at the beginning of this section, future studies on cyber-dating abuse could focus on the role of stigma, stereotypes, and self-blame among young people and particularly young women, to provide a specific recommendation on how the awareness of young people can be enhanced to reduce their self-blame and mechanisms to encourage them to seeking help.

As indicated in the findings’ section, there is a connection between online and offline IPV; nevertheless, there are some precipitating factors that make online abuse more elusive than in-person abuse, including the nebulous nature of cyber-dating abuse, being spaceless and the difficulties of distinguishing when the abuse occurs and the process of reporting. For instance, Barter (2018) indicates that controlling behavior can be normalized in young people’s relationships, particularly with a male partner. Social media platforms, however, provided an opportunity for constant checking, monitoring, and control and fueled some behaviors among young people, such as jealousy, to become more vicious and lead to aggressive and abusive behaviors. Also, the capacity to capture screenshots of private online images and distribute them easily and quickly is often an act perpetrated by young men and boys. The pattern of abuse and how it should be responded to needs further exploration, focusing on how the safety of digital tools and online platforms can be improved collectively with young people, social workers, IT developers, and police forces.

Although the scoping review provides useful insights into young people’s experiences of cyber-dating abuse, from a gender lens, caution needs to be exercised in the interpretation of the findings in light of two key gaps in the existing research base. Firstly, all studies, bar one, reported on cyber-dating abuse in Western sociocultural contexts, namely, European and North American countries and Australia. There were no studies from African or South-East Asian countries. As such, the reported similarities across studies may reflect the Western-centric nature of the research base rather than a level of homogeneity in young people’s experience of cyber-dating abuse across the globe. Although, unsurprisingly, Western experiences and perspectives dominate the existing literature, the dearth of research on cyber-dating abuse across the globe is concerning, given the omnipresence of information and communication technologies worldwide. It is notable that data are not available from countries and regions (e.g., Southeast Asia, Middle East, and Africa) that often arguably represent societies with more conservative gender roles (Afrouz et al., 2023) and/or have lower ranks in gender inequality index (UNDP, 2022). In some of those contexts, romantic relationships before marriage are restricted or considered against the law (Motamedi et al., 2016), and therefore, young people’s experiences, coping mechanisms, and access to support systems might be very different from European and North American contexts. In addition to the gap in literature among countries, research has been limited in exploring young people with diverse identities. Further research can provide a broader understanding of cyber-dating abuse among young women and girls across different social locations and identities, such as race, ethnicity, sexual orientation, gender identities, and young women with disabilities.

Secondly, there was little consistency across the studies in their definition of adolescents and young people as a research population. Some studies were more narrowly focused on what is generally regarded as the adolescent years (13–19 years), whereas others include the perspectives and experiences of young people in late childhood (11–12 years), adolescence, and young adulthood (20–24 years). These definitional issues impact how the various studies cohere as a body of research on young people’s experiences of cyber-dating abuse. This inconsistency also made it difficult to conclude and distinguish the specific impacts of cyber-dating abuse on adolescents below 18 years. As this group might be more vulnerable to cyber-dating abuse and need more support, further research will be required to understand the unique impacts of cyber-dating abuse on under-18s in order to provide relevant recommendations for parents/guardians and indicate the tool for schools for prevention and early intervention programs. Further research can explore the role of parents in developing and strengthening their contribution to enhance protective factors while minimizing risk among young people (Taket & Crisp, 2018).

Thirdly, while the findings of this paper indicate that gender norms can provide a context for abusing and controlling women in the digital world, the methodology and study samples of included papers tended to adopt a heterosexual and cisgender lens. The voices and experiences of the LGPTQI+ population have been missing in the existing literature, which highlights a significant gap in this research area. More research is needed to provide a picture of cyber-dating abuse among gender and sexual minority young people.

In addition to the gap in the literature, the process of this scoping review has had a few limitations that need to be highlighted. Only peer-reviewed journal articles were included to ensure consistency, research integrity, and quality. Some reports that are published as gray literature might have been missed; however, authors could not identify all international websites and platforms to access those recourses. Only included English papers were included; although the first author searched for Persian language papers, only English papers were identified as relevant. International studies can include other languages to provide insights into cyber-dating violence within different nations and communities. Many of the included studies in this review also reported data before the COVID-19 pandemic, while the communication and online world has been transformed post-COVI9-19 era, which can be further explored within future reviews.

## Conclusion and Recommendations

This study aimed to identify the experiences of young people of cyber-dating abuse to identify patterns, impacts, and consequences of cyber-dating abuse among adolescents. The findings of this paper indicated the complex nature of male domination, systematic control, and the new capacity that digital technology provides for gender inequality. Online platforms and digital technology have potentially exacerbated the monitoring, control, and surveillance of young women, often by young men. At the same time, the nature of cyber-dating abuse is elusive, and there is a blurring of the line between romantic enmeshment in cyberspace and technology-facilitated abuse within intimate relationships. Future research can address this gap by exploring the breadth of young people’s definitions and understandings of cyber-dating abuse in order to tailor prevention programs that respond to young people’s diverse information needs.

As the review identified, young women and girls were more likely than their male counterparts to experience negative psychosocial impacts, including a tendency to blame themselves for their victimization. A systematic approach to early intervention and prevention through the primary and secondary schools’ curricula is required. For example, specific prevention programs can be developed to heighten young women’s awareness of early signs of cyber-dating and the gendered patterns of abuse. Prevention programs can also be developed to sensitize both girls and boys to their rights to privacy and informed consent in the online space. In addition, primary prevention and intervention programs ought to be tailored to reflect the patterns of online dating abuse to deter digital technology and online platforms from becoming tools for cyber abuse. Further initiatives can focus on young people’s education, promoting digital literacy, and respectful and responsible online behaviors are crucial strategies for combating cyber abuse.

This review indicated how digital technology played a significant role in providing a tool for perpetrators and exacerbated this form of abuse; nevertheless, cyber-dating abuse reflects the gender inequality in an intimate relationship which is a driver for gendered-based abuse. Furthermore, this finding reinforces the gendered nature and impacts of cyber-dating abuse and the need for early intervention for gender equity and respectful relationships within the community and through digital technology. Developing prevention programs are recommended to challenge social norms on gender inequality, male power, and victim blaming within communities and relevant platforms (Barter, 2018). For example, prevention programs in schools and public campaigns within local communities and online social media can support young people to develop a consciousness and attain the required knowledge to be able to recognize cyber-dating and other forms of abuse. Positive bystander programs can also support young men and boys to develop an awareness of and shift harmful gender stereotypes and victim-blaming norms (see Taket & Crisp, 2018).

The relevant training and policy development can review the current IPV support networks and how these can be developed to respond effectively to this form of abuse. In addition, it is crucial that training is accessible and available for different groups of young people. Young people also need to be informed of the available support, and such support should be accessible to young people. This becomes particularly critical given that young women experiencing cyber-dating abuse were least likely to access formal services and support.

A summary of implications for policy, research and practice is presented in Table 4.

**Table 4.** Implications for Policy, Practice, and Research.

Further research can provide a broader understanding of cyber-dating abuse among young women and girls across different countries, social locations, and identities, such as race, ethnicity, sexual orientation, gender identities, and young women with disabilities.
Further research is required to understand the unique impacts of cyber-dating abuse on under-18s as they might be more vulnerable to cyber-dating abuse, which could provide relevant recommendations for parents/guardians and indicate the tool for schools for prevention and early intervention programs.
Age-specific education for young people, particularly young women, can play a significant role in identifying the signs of cyber-dating violence and how they should be reported when needed.
Public awareness and training programs should be further available for young people to understand different forms of abuse that occur through technology, such as monitoring, financial and emotional violence, harassment, and stalking.
Respectful relationship programs in schools and higher education are necessary to prevent this form of abuse.
Prevention and intervention programs should include parents and schools to strengthen their contribution to enhancing protective factors while minimizing risk among young people.
Further training is needed to enhance police and services’ understanding of young people’s experiences of cyber-dating abuse and how they can address this form of abuse.

## References

[bibr1-15248380241227457] Afrouz R . (2021). The nature, patterns and consequences of technology-facilitated domestic abuse: A scoping review. Trauma, Violence, & Abuse, 24(2), 15248380211046752. 10.1177/15248380211046752 34582729

[bibr2-15248380241227457] Afrouz R. Crisp B. R. Taket A. (2023). Afghan women perceptions of gender roles, possibilities and barriers to change after settlement in Australia: A qualitative study. Qualitative Social Work, 22(3), 569–586. 10.1177/147332502210767

[bibr3-15248380241227457] Alsawalqa R. O. (2021). Evaluating female experiences of electronic dating violence in Jordan: motivations, consequences, and coping strategies. Frontiers in Psychology, 12, 1–13. 10.3389/fpsyg.2021.719702 PMC8669046 34916986

[bibr4-15248380241227457] Arksey H. O’Malley L. (2005). Scoping studies: Towards a methodological framework. International Journal of Social Research Methodology, 8(1), 19–32. 10.1080/1364557032000119616

[bibr5-15248380241227457] Baker C. K. Carreño P. K. (2016). Understanding the role of technology in adolescent dating and dating violence. Journal of Child and Family Studies, 25(1), 308–320.

[bibr6-15248380241227457] Baker C. K. Helm S. (2010). Pacific youth and shifting thresholds: Understanding teen dating violence in Hawai ‘i. Journal of School Violence, 9(2), 154–173. 10.1080/15388220903585879

[bibr7-15248380241227457] Barter C. (2018). Violence and abuse in young people’s intimate relationships: Interface of gender, prevalence, impact and implications for prevention. In Taket A. Crisp B. R. (Eds.), Eliminating gender-based violence (1st ed., pp. 68–82). Routledge.

[bibr8-15248380241227457] Belotti F. Ieracitano F. Donato S. Comunello F. (2022). Towards “romantic media ideologies”: Digital dating abuse seen through the lens of social media and/or dating in teenage narratives. The Communication Review, 25(1), 30–53. 10.1080/10714421.2022.2033576

[bibr9-15248380241227457] Borrajo E. Gámez-Guadix M. Calvete E. (2015). Cyber dating abuse: Prevalence, context, and relationship with offline dating aggression. Psychological Reports, 116(2), 565–585. 10.2466/21.16.PR0.116k22w4 25799120

[bibr10-15248380241227457] Braun V. Clarke V. (2019). Reflecting on reflexive thematic analysis. Qualitative Research in Sport, Exercise and Health, 11(4), 589–597.

[bibr11-15248380241227457] Brooks A. Hesse-Biber S. N. (2011). An invitation to feminist research. In Hesse-Biber S. N. (Ed.), Feminist research practice: A primer (Vol. 1, pp. 2–24). SAGE.

[bibr12-15248380241227457] Brown C. Flood M. Hegarty K. (2020). Digital dating abuse perpetration and impact: The importance of gender. Journal of Youth Studies, 25(2), 193–208.

[bibr13-15248380241227457] Caridade S. Braga T. Borrajo E. (2019). Cyber dating abuse (CDA): Evidence from a systematic review. Aggression and Violent Behavior, 48, 152–168. 10.1016/j.avb.2019.08.018

[bibr14-15248380241227457] Caridade S. M. M. Braga T. (2020). Youth cyber dating abuse: A meta-analysis of risk and protective factors. Cyberpsychology: Journal of Psychosocial Research on Cyberspace, 14(3), 1–26. 10.5817/CP2020-3-2

[bibr15-15248380241227457] Cocker C. Hafford-Letchfield T. (2022). Rethinking feminist theories for social work practice. In Cocker C. Hafford-Letchfield T. (Eds.), Rethinking feminist theories for social work practice (pp. 1–14). Springer.

[bibr16-15248380241227457] Critical Appraisal Skills Programme. (2017). CASP checklist, Oxford, UK. Retrieved June 5, 2017, from http://www.casp-uk.net/casp-tools-checklists

[bibr17-15248380241227457] Dick R. N. McCauley H. L. Jones K. A. Tancredi D. J. Goldstein S. Blackburn S. Monasterio E. James L. Silverman J. G. Miller E. (2014). Cyber dating abuse among teens using school-based health centers. Pediatrics, 134(6), e1560–e1567. 10.1542/peds.2014-0537 25404724

[bibr18-15248380241227457] Draucker C. B. Martsolf D. S. (2010). The role of electronic communication technology in adolescent dating violence. Journal of Child and Adolescent Psychiatric Nursing, 23(3), 133–142. 10.1111/j.1744-6171.2010.00235.x 20796096

[bibr19-15248380241227457] Fernández-Antelo I. Cuadrado-Gordillo I. Martín-Mora Parra G. (2020). Synergy between acceptance of violence and sexist attitudes as a dating violence risk factor. International Journal of Environmental Research and Public Health, 17(14), 5209. 10.3390/ijerph17145209 32707658 PMC7400527

[bibr20-15248380241227457] Fernet M. Hébert M. Brodeur G. Guyon R. Lapierre A. (2023). Youth’s experiences of cyber violence in intimate relationships: A matter of love and trust. Journal of Child Sexual Abuse, 32(3), 296–317. 10.1080/10538712.2023.2167678 36662508

[bibr21-15248380241227457] Flynn A. Cama E. Powell A. Scott A. J. (2022). Victim-blaming and image-based sexual abuse. Journal of Criminology, 56(1), 7–25. 10.1177/26338076221135327

[bibr22-15248380241227457] Flynn A. Powell A. Hindes S. (2021). Technology-facilitated abuse: A survey of support services stakeholders. ANROWS. https://www.anrows.org.au/publication/technology-facilitated-abuse-a-survey-of-support-services-stakeholders/

[bibr23-15248380241227457] George A. Harris B. (2014). Landscapes of violence: Women surviving family violence in regional and rural Victoria. Deakin University. Retrieved October 24, 2020, from https://www.deakin.edu.au/__data/assets/pdf_file/0003/287040/Landscapes-of-Violence-online-pdf-version.pdf

[bibr24-15248380241227457] Goldstein A. Flicker S. (2020). “Some Things Just Won’t Go Back”: Teen Girls’ Online Dating Relationships during COVID-19. Girlhood Studies, 13(3), 64–78.

[bibr25-15248380241227457] Grant M. J. Booth A. (2009). A typology of reviews: An analysis of 14 review types and associated methodologies. Health Information & Libraries Journal, 26(2), 91–108. 19490148 10.1111/j.1471-1842.2009.00848.x

[bibr26-15248380241227457] Harris B. (2018). Spacelessness, spatiality and intimate partner violence Technology-facilitated abuse, stalking and justice administration. In Fitz-Gibbon K. Walklate S. McCulloch J. Maher J. (Eds.), Intimate partner violence, risk and security (pp. 52–70). Routledge.

[bibr27-15248380241227457] Hellevik P. M. (2019). Teenagers’ personal accounts of experiences with digital intimate partner violence and abuse. Computers in Human Behavior, 92, 178–187.

[bibr28-15248380241227457] Henry N. Powell A. (2018). Technology-facilitated sexual violence: A literature review of empirical research. Trauma, Violence, & Abuse, 19(2), 195–208. 10.1177/1524838016650189 27311818

[bibr29-15248380241227457] Hertlein K. M. (2021). The weaponized web: How internet technologies fuel intimate partner violence. International Journal of Systemic Therapy, 32(3), 171–193.

[bibr30-15248380241227457] Hesse-Biber S. N. Leavy P. (2011). Feminist research practice: A primer (Vol. 1). SAGE.

[bibr31-15248380241227457] Howard D. E. Debnam K. J. Strausser A. (2019). “I’m a stalker and proud of it”: Adolescent girls’ perceptions of the mixed utilities associated with internet and social networking use in their dating relationships. Youth and Society, 51(6), 773–792.

[bibr32-15248380241227457] Johnson R. B. Onwuegbuzie A. J. Turner L. A. (2007). Toward a d﻿efinition of m﻿ixed m﻿ethods research. Journal of Mixed Methods Research, 1(2), 112–133. 10.1177/1558689806298224

[bibr33-15248380241227457] Kennedy A. C. Prock K. A. (2018). “I still feel like I am not normal”: A review of the role of stigma and stigmatization among female survivors of child sexual abuse, sexual assault, and intimate partner violence. Trauma, Violence, & Abuse, 19(5), 512–527. 10.1177/1524838016673601 27803311

[bibr34-15248380241227457] Kulkarni S. J. Porter A. M. Mennick A. Gil-Rivas V. (2019). “I feel like. . . their relationship is based on the media”: Relationship between media representation and adolescents’ relationship knowledge and expectations. The Journal of Primary Prevention, 40(5), 545–560. 10.1007/s10935-019-00565-0 31571031 PMC13390789

[bibr35-15248380241227457] Lachapelle M. Fernet M. Hébert M. Guyon R. (2021). A mixed methods approach exploring risk factors associated with cyber dating victimization and resilience in adolescents and emerging adults. Journal of Aggression, Maltreatment & Trauma, 31(5), 589–608. 10.1080/10926771.2021.1994499

[bibr36-15248380241227457] Linares R. Aranda M. García-Domingo M. Amezcua T. Fuentes V. Moreno-Padilla M. (2021). Cyber-dating abuse in young adult couples: Relations with sexist attitudes and violence justification, smartphone usage and impulsivity. PLoS One, 16(6), 1–19. 10.1371/journal.pone.0253180 PMC8216513 34153073

[bibr37-15248380241227457] Lu Y. Van Ouytsel J. Temple J. R. (2021). In-person and cyber dating abuse: A longitudinal investigation. Journal of Social and Personal Relationships, 38(12), 3713–3731. 36382139 10.1177/02654075211065202 PMC9645533

[bibr38-15248380241227457] Lucero J. L. Weisz A. N. Smith-Darden J. Lucero S. M. (2014). Exploring gender differences: Socially interactive technology use/abuse among dating teens. Affilia, 29(4), 478–491.

[bibr39-15248380241227457] Lundgren R. Amin A. (2015). Addressing intimate partner violence and sexual violence among adolescents: Emerging evidence of effectiveness. Journal of Adolescent Health, 56(1), S42–S50. 10.1016/j.jadohealth.2014.08.012 25528978

[bibr40-15248380241227457] Mandau M. B. H. (2021). “Snaps”, “screenshots”, and self-blame: A qualitative study of image-based sexual abuse victimization among adolescent Danish girls. Journal of Children and Media, 15(3), 431–447.

[bibr41-15248380241227457] Morelli M. Bianchi D. Chirumbolo A. Baiocco R. (2018). The cyber dating violence inventory. Validation of a new scale for online perpetration and victimization among dating partners. European Journal of Developmental Psychology, 15(4), 464–471. 10.1080/17405629.2017.1305885

[bibr42-15248380241227457] Motamedi M. Merghati-Khoei E. Shahbazi M. Rahimi-Naghani S. Salehi M. Karimi M. Hajebi A. Khalajabadi-Farahani F. (2016). Paradoxical attitudes toward premarital dating and sexual encounters in Tehran, Iran: A cross-sectional study. Reproductive health, 13(1), 1–10. 10.1186/s12978-016-0210-4 27576489 PMC5006512

[bibr43-15248380241227457] Murray A. (2019). Teen dating violence: Old disease in a new world. Clinical Pediatric Emergency Medicine, 20(1), 25–37. 10.1016/j.cpem.2019.02.001

[bibr44-15248380241227457] Peskin M. F. Markham C. M. Shegog R. Temple J. R. Baumler E. R. Addy R. C. Hernandez B. Cuccaro P. Gabay E. K. Thiel M. (2017). Prevalence and correlates of the perpetration of cyber dating abuse among early adolescents. Journal of Youth and Adolescence, 46(2), 358–375. 10.1007/s10964-016-0568-1 27665278 PMC13011686

[bibr45-15248380241227457] Pham M. T. Rajić A. Greig J. D. Sargeant J. M. Papadopoulos A. McEwen S. A. (2014). A scoping review of scoping reviews: Advancing the approach and enhancing the consistency. Research Synthesis Methods, 5(4), 371–385. 26052958 10.1002/jrsm.1123 PMC4491356

[bibr46-15248380241227457] Powell A. Henry N. (2019). Technology-facilitated sexual violence victimization: Results from an online survey of Australian adults. Journal of Interpersonal Violence, 34(17), 3637–3665. 27697966 10.1177/0886260516672055

[bibr47-15248380241227457] Powell A. Scott A. J. Henry N. (2018). Digital harassment and abuse: Experiences of sexuality and gender minority adults. European Journal of Criminology, 17(2), 199–223. 10.1177/1477370818788006

[bibr48-15248380241227457] Rajah V. Osborn M. (2020). Understanding women’s resistance to intimate partner violence: A scoping review. Trauma, Violence, & Abuse, 23(5), 1373–1387. 10.1177/1524838019897345 31920172

[bibr49-15248380241227457] Reed L. A. Conn K. Wachter K. (2020). Name-calling, jealousy, and break-ups: Teen girls’ and boys’ worst experiences of digital dating. Children and Youth Services Review, 108, 104607. 10.1016/j.childyouth.2019.104607

[bibr50-15248380241227457] Reed L. A. Tolman R. M. Ward L. M. (2017). Gender matters: Experiences and consequences of digital dating abuse victimization in adolescent dating relationships. Journal of Adolescence, 59, 79–89. 10.1016/j.adolescence.2017.05.015 28582653

[bibr51-15248380241227457] Reyes H. Foshee V. A. Niolon P. H. Reidy D. E. Hall J. E. (2016). Gender role attitudes and male adolescent dating violence perpetration: Normative beliefs as moderators. Journal of Youth and Adolescence, 45(2), 350–360. 25831994 10.1007/s10964-015-0278-0 PMC4592366

[bibr52-15248380241227457] Rodríguez-deArriba M.-L. Nocentini A. Menesini E. Sánchez-Jiménez V. (2021). Dimensions and measures of cyber dating violence in adolescents: A systematic review. Aggression and Violent Behavior, 58, 1–10. 10.1016/j.avb.2021.101613

[bibr53-15248380241227457] Rueda H. A. Brown M. L. Geiger J. M. (2020). Technology and dating among pregnant and parenting youth in residential foster care: A mixed qualitative approach comparing staff and adolescent perspectives. Journal of Adolescent Research, 35(4), 521–545.

[bibr54-15248380241227457] Rueda H. A. Lindsay M. Williams L. R. (2015). “She posted It on facebook” Mexican American adolescents’ experiences with technology and romantic relationship conflict. Journal of Adolescent Research, 30(4), 419–445.

[bibr55-15248380241227457] Sardinha L. Maheu-Giroux M. Stöckl H. Meyer S. R. García-Moreno C. (2022). Global, regional, and national prevalence estimates of physical or sexual, or both, intimate partner violence against women in 2018. The Lancet, 399(10327), 803–813. 10.1016/S0140-6736(21)02664-7 PMC8885817 35182472

[bibr56-15248380241227457] Sen R. (2016). Not all that is solid melts into air? Care-experienced young people, friendship and relationships in the “digital age”. The British Journal of Social Work, 46(4), 1059–1075. 27559214 10.1093/bjsw/bcu152 PMC4986133

[bibr57-15248380241227457] Smith K. Cénat J. M. Lapierre A. Dion J. Hébert M. Côté K. (2018). Cyber dating violence: Prevalence and correlates among high school students from small urban areas in Quebec. Journal of Affective Disorders, 234, 220–223. 10.1016/j.jad.2018.02.043 29544168

[bibr58-15248380241227457] Stanley N. Barter C. Wood M. Aghtaie N. Larkins C. Lanau A. Överlien C. (2018). Pornography, sexual coercion and abuse and sexting in young people’s intimate relationships: A European study. Journal of Interpersonal Violence, 33(19), 2919–2944. 10.1177/0886260516633204 26951609

[bibr59-15248380241227457] Stöckl H. March L. Pallitto C. Garcia-Moreno C. (2014). Intimate partner violence among adolescents and young women: Prevalence and associated factors in nine countries: A cross-sectional study. BMC Public Health, 14(1), 1–14. 25059423 10.1186/1471-2458-14-751 PMC4133076

[bibr60-15248380241227457] Stonard K. E. (2020). “Technology was designed for this”: Adolescents’ perceptions of the role and impact of the use of technology in cyber dating violence. Computers in Human Behavior, 105, 106211.

[bibr61-15248380241227457] Stonard K. E. Bowen E. Walker K. Price S. A. (2017). “They’ll always find a way to get to you”: Technology use in adolescent romantic relationships and its role in dating violence and abuse. Journal of Interpersonal Violence, 32(14), 2083–2117. 26065711 10.1177/0886260515590787

[bibr62-15248380241227457] Taket A. Crisp B. R. (2018). Eliminating gender-based violence. Routledge.

[bibr63-15248380241227457] Taylor B. G. Mumford E. A. (2016). A national descriptive portrait of adolescent relationship abuse: Results from the National Survey on Teen Relationships and Intimate Violence. Journal of Interpersonal Violence, 31(6), 963–988. 10.1177/0886260514564070 25548142

[bibr64-15248380241227457] Temple J. R. Choi H. J. Brem M. Wolford-Clevenger C. Stuart G. L. Peskin M. F. Elmquist J. (2016). The temporal association between traditional and cyber dating abuse among adolescents. Journal of Youth and Adolescence, 45(2), 340–349. 10.1007/s10964-015-0380-3 26525389 PMC4713259

[bibr65-15248380241227457] Towler A. Eivers A. Frey R. (2020). Warning signs of partner abuse in intimate relationships: Gender differences in young adults’ perceptions of seriousness. Journal of Interpersonal Violence, 35(7–8), 1779–1802. 29294689 10.1177/0886260517696869

[bibr66-15248380241227457] UNDP. (2022). Gender inequality index. Retrieved October 14, 2022, from https://hdr.undp.org/data-center/thematic-composite-indices/gender-inequality-index#/indicies/GII

[bibr67-15248380241227457] UNICEF. (2022). Adolescents. Retrieved September 29, 2022, from https://data.unicef.org/topic/adolescents/overview/#:~:text=Defined%20by%20the%20United%20Nations,the%20Rights%20of%20the%20Child

[bibr68-15248380241227457] Van Ouytsel J. Walrave M. Ponnet K. Willems A.-S. Van Dam M . (2019). Adolescents’ perceptions of digital media’s potential to elicit jealousy, conflict and monitoring behaviors within romantic relationships. Cyberpsychology-Journal of Psychosocial Research on Cyberspace, 13(3), UNSP-3.

[bibr69-15248380241227457] Wake A. D. Kandula U. R. (2022). The global prevalence and its associated factors toward domestic violence against women and children during COVID-19 pandemic—“The shadow pandemic”: A review of cross-sectional studies. Women’s Health, 18, 1–13. 10.1177/17455057221095536 PMC9024155 35441537

[bibr70-15248380241227457] Weathers M. R. Canzona M. R. Fisher C. L. (2019). Digital media as a context for dating abuse: Connecting adaptive and maladaptive coping strategies to young adult women’s well-being. Affilia, 34(3), 325–345. 10.1177/0886109919832005

[bibr71-15248380241227457] Weathers M. R. Hopson M. C. (2015). “I Define What Hurts Me”: A co-cultural theoretical analysis of communication factors related to digital dating abuse. Howard Journal of Communications, 26(1), 95–113. 10.1080/10646175.2015.988475

[bibr72-15248380241227457] Williams S. R. Afrouz R. Vassos S. (2023). Exploring rural and regional social workers’ perceptions and practices of technology-facilitated domestic abuse. Australian Social Work, 76(2), 231–244. 10.1080/0312407X.2021.1985547

[bibr73-15248380241227457] World Health Organization. (2014). Global status report on violence prevention 2014. Retrieved February 15, 2023 from http://www.who.int/violence_injury_prevention/violence/status_report/2014/en/

[bibr74-15248380241227457] Zweig J. M. Dank M. Yahner J. Lachman P. (2013). The rate of cyber dating abuse among teens and how it relates to other forms of teen dating violence. Journal of Youth and Adolescence, 42(7), 1063–1077. 23412689 10.1007/s10964-013-9922-8

[bibr75-15248380241227457] Zweig J. M. Lachman P. Yahner J. Dank M. (2014). Correlates of cyber dating abuse among teens. Journal of Youth and Adolescence, 43(8), 1306–1321. 24198083 10.1007/s10964-013-0047-x

